# Prospective, double-blind, randomized, placebo-controlled phase III study evaluating efficacy and safety of octagam 10% in patients with dermatomyositis (“ProDERM Study”)

**DOI:** 10.1097/MD.0000000000023677

**Published:** 2021-01-08

**Authors:** Rohit Aggarwal, Christina Charles-Schoeman, Joachim Schessl, Mazen M. Dimachkie, Irene Beckmann, Todd Levine

**Affiliations:** a University of Pittsburgh School of Medicine, Pittsburgh, PA; b University of California, Los Angeles, CA; c Friedrich-Baur-Institute, Department of Neurology, Ludwig-Maximilians University of Munich, Munich, Germany; d University of Kansas Medical Center, Kansas City, KS; e Octapharma Pharmazeutika Produktionsges.m.b.H., Vienna, Austria; f Department of Neurology, Phoenix Neurological Associates, Ltd., Phoenix, AZ.

**Keywords:** dermatomyositis, idiopathic inflammatory myopathy, immunomodulation, intravenous immunoglobulin (IVIg), octagam, ProDERM study, randomized controlled trial

## Abstract

**Introduction:**

Dermatomyositis (DM) is an inflammatory myopathy characterized by distinct skin manifestations and muscle weakness. Intravenous immunoglobulin (IVIg) has been used off-label as adjuvant therapy in DM, but is not indicated for DM, due to lack of proven efficacy in a large randomized controlled trial. The objective of the ProDERM (Progress in DERMatomyositis) study was to evaluate the efficacy, safety and long-term tolerability of IVIg (Octagam 10%) in patients with DM in a randomized, placebo-controlled, double-blind, Phase III study.

**Methods:**

Adult patients with active DM who were continuing standard therapy at a stable dose were eligible for this study. Patients were randomized 1:1 to receive either 2 g/kg of IVIg or placebo, administered every 4 weeks until week 16 (First Period). Patients were switched to the alternate treatment if they showed clinical deterioration in the First Period. After response assessment at week 16, all patients on placebo and those without deterioration on IVIg entered the open-label Extension Period, receiving 2 g/kg IVIg every 4 weeks for 24 weeks.

**Results:**

The primary efficacy endpoint was the proportion of responders in the IVIg vs placebo arm at week 16, where response was defined per 2016 ACR/EULAR Myositis Response Criteria of at least minimal improvement [Total Improvement Score (TIS) ≥20] and without deterioration at 2 consecutive visits up to week 16. TIS consists of composite response criteria, combining weighted improvement in 6 core set measures (CSMs), Global Disease Activity (Physician and Patient), manual muscle testing-8 (MMT-8), Health Assessment Questionnaire, extra-muscular disease activity, and muscle enzymes. Secondary endpoints included the mean change in individual CSMs, time to improvement in TIS, time to confirmed deterioration in the First Period, and the overall proportion of patients with deteriorations. Adverse events, including infusion reactions and thromboembolic events, were recorded.

**Conclusions:**

The ProDERM study was the first to assess the long-term efficacy and safety of IVIg (Octagam 10%) in a placebo-controlled, blinded, randomized trial in DM. The study aimed to inform on the use of IVIg in the treatment of DM, and results are expected in Q3 2020.

**ClinicalTrials.gov Identifier:**

NCT02728752.

## 1 Introduction

Adult dermatomyositis (DM) is an idiopathic inflammatory myopathy characterized by distinct skin manifestations and by chronic inflammation of striated muscle, predominately in proximal muscles, leading to progressive muscle weakness. Although the precise pathogenesis is unknown, DM likely results from autoimmune processes.^[1]^ Despite significant morbidity and mortality associated with DM, there are currently no therapies approved in patients with DM by the US or European regulatory authorities based on evidence from randomized controlled trials. However, off-label immunosuppressive and immunomodulating therapy use is widespread. Several European and American national guidelines for the treatment of DM recommend intravenous immunoglobulin (IVIg) as adjuvant treatment with continuation of immunosuppressive therapy and corticosteroids.^[2–4]^

Despite several reports of observational and retrospective studies assessing the beneficial effects of IVIg in DM,^[1,5]^ only one placebo-controlled clinical trial, dating back more than 25 years, has been published.^[6]^ In this small trial, 15 patients with refractory DM were treated with a dose of 2.0 g/kg IVIg or placebo for 12 weeks, with the option to cross over to the other therapy for 3 additional months.^[6]^ A total of 12 patients received IVIg, of whom 9 patients had a major improvement to nearly normal function.^[6]^ Patients treated with IVIg had a significant improvement in muscle strength and neuromuscular symptoms in contrast to patients on placebo. While this study, and other retrospective studies, show that patients with DM benefit from IVIg treatment, a large randomized, placebo-controlled study applying validated and robust outcome measures is warranted.^[7,8]^

Octagam 10% (Octapharma AG, Lachen, Switzerland) is a liquid intravenous polyvalent IVIg preparation, prepared from human plasma containing mainly highly purified normal human immunoglobulin G. The aim of the ProDERM (“Progress in DERMatomyositis”) study (NCT02728752) was to investigate the efficacy, safety and tolerability of high-dose IVIg (2.0 g/kg, Octagam 10%) in DM patients. Furthermore, as many patients with DM may need lifelong treatment, data from the ProDERM study will provide important new information on the long-term efficacy and safety of high-dose IVIg in patients with DM. Here, we report the unique design and features of the ProDERM study.

## 2 Methods

### 2.1 Design

The ProDERM study was a prospective, parallel group, double-blind, randomized, placebo-controlled, multicenter Phase III study to assess the efficacy, safety and tolerability of IVIg (Octagam 10%) in the treatment of DM. The study started enrollment on February 27, 2017, and was clinically completed with the last patient visit on November 5, 2019.

**Figure 1:**
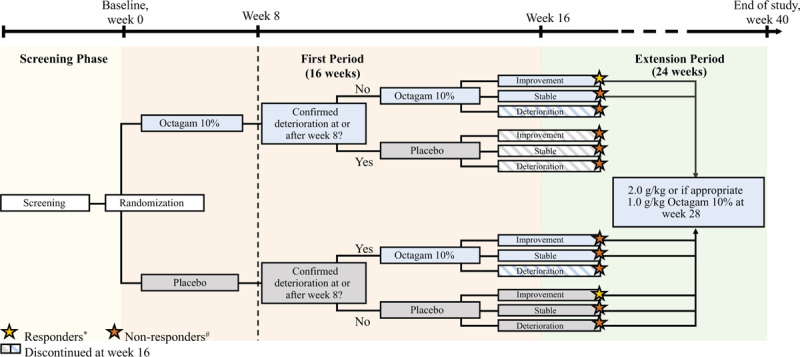
Overview of study design. ^∗^Patients with a total improvement score (TIS) ≥20 at week 16 and no prior confirmed deterioration up to and including week 16. ^#^Patients discontinuing from the study due to confirmed deterioration and patients with no response.

Patient consent was obtained prior to entry into a Screening Period in which eligibility criteria were assessed. The initial efficacy evaluation period, the First Period, began after randomization (Baseline, week 0) to either 2 g/kg of IVIg or placebo treatment. The patients received their respective treatment at 4-week intervals for 4 infusion cycles (at weeks 0, 4, 8, and 12). The First Period ended at week 16, at which point the patients either discontinued the study or continued to the open-label Extension Period, lasting for an additional 24 weeks, during which they received open-label IVIg for another 6 cycles, until week 40 (Final Assessment visit). An overview of the study design is shown in Figure 1.

During the First Period, visits were scheduled every 4 weeks starting at Baseline and ending at week 16. Treatment was administered at each visit, and after each infusion the Wells probability score for deep vein thrombosis (DVT) and pulmonary embolisms (PEs) was assessed to evaluate risk for thromboembolic events (TEEs). At every visit during the First Period, at week 28 and at the Final Assessment visit (week 40), Core Set Measurements (CSMs) and the Cutaneous Dermatomyositis Disease Area and Severity Index (CDASI) were evaluated. The short form (36) health survey (SF-36v2) was assessed at Baseline, at the end of the First Period and at the Final Assessment visit. A direct Coombs test was performed at Baseline, at the end of the First Period, week 28 and at the Final Assessment visit. Immunoglobulin G (IgG) trough level concentrations were measured at each visit prior to placebo or IVIg administration in order to potentially correlate the IgG levels with the disease activity and responder classification. A biorepository blood sample was collected in order to investigate potential biomarkers of disease activity (e.g., myositis-specific antibodies, cytokine, chemokine or monoclonal antibody changes).

An independent data monitoring committee, composed of 3 experts in the fields of immunology, rheumatology and dermatology, and one statistician, regularly reviewed relevant safety data, such as the occurrences of TEEs, and gave advice on the continuation, modification or termination of the study. The study was conducted in accordance with the ethical principles laid down in the Declaration of Helsinki and in compliance with good clinical practice (GCP) guidelines. The study was approved by the relevant Independent Ethics Committees or Institutional Review Boards, as applicable, prior to each sites initiation. The study protocol was approved by the relevant Independent Ethics Committees and/or Institutional Review Boards at each of the participating centers in the United States, Canada, Czechia, Germany, Hungary, the Netherlands, Poland, Romania, Russian Federation and Ukraine. A complete list of all participating study centers can be found on https://clinicaltrials.gov/ct2/show/NCT02728752. The study was sponsored by Octapharma Pharmazeutika Produktionsges.m.b.H. Oberlaaer Str. 235, 1100 Vienna, Austria.

### 2.2 Patient population

Enrollment of a minimum of 94 adult patients with definite or probable DM according to the Bohan and Peter criteria was planned.^[9,10]^ Fifty five sites from 10 countries worldwide were planned to participate in the study. Full inclusion and exclusion criteria are shown in Table 1. The study included patients with active DM who were receiving standard care for DM. Active DM was determined by consensus of an independent adjudication committee, which consisted of 4 permanent members who had experience in the fields of neurology, rheumatology and/or dermatology.

**Table 1:** Inclusion and exclusion criteria of study.

Inclusion criteria	
1.	Patients with diagnosis of definite or probable DM according to the Bohan and Peter criteria
2.	Patients under treatment with corticosteroids and/or maximally two immune-suppressants and being on stable therapy for at least four weeks OR patients with previous failure of response or previous intolerance to corticosteroid and at least one additional immunosuppressive drug, and with steroid/immunosuppressive drugs washed out
3.	Patients with active disease, assessed and agreed upon by an independent adjudication committee
4.	MMT-8 score <142, with at least two other abnormal CSM, VAS of patient global activity ≥2 cm, physician's global disease activity ≥2 cm, extra-muscular activity ≥2 cm; at least one muscle enzyme >1.5 times upper limit of normal, HAQ ≥0.25)
5.	Males or females ≥18 to <80 years of age
6.	Voluntarily given, fully informed written consent obtained from patient before any study-related procedures are conducted
7.	Patient must be capable to understand and comply with the relevant aspects of the study protocol
Exclusion criteria	
1.	Cancer-associated myositis, defined as the diagnosis of myositis within two years of the diagnosis of cancer (except basal or squamous cell skin cancer or carcinoma in situ of the cervix that has been excised and cured and at least one or five years, respectively, have passed since excision)
2.	Evidence of active malignant disease or malignancies diagnosed within the previous five years (including hematological malignancies and solid tumors) or breast cancer diagnosed within the previous 10 years
3.	Patients with overlap myositis (except for overlap with Sjögren's syndrome), connective tissue disease associated DM, inclusion body myositis, polymyositis, juvenile dermatomyositis or drug-induced myopathy
4.	Patients with immune-mediated necrotizing myopathy with absence of typical DM rash
5.	Patients with generalized, severe musculoskeletal conditions other than DM that prevent a sufficient assessment of the patient by the physician
6.	Patients who have received IgG treatment within the last six months before enrollment
7.	Patients who received blood or plasma-derived products (other than IgG) or plasma exchange within the last three months before enrollment
8.	Patients starting or planning to start a physical therapy-directed exercise regimen during the trial
9.	Cardiac insufficiency (New York Heart Association III/IV), cardiomyopathy, significant cardiac dysrhythmia requiring treatment, unstable or advanced ischemic heart disease
10.	Severe liver disease, with signs of ascites and hepatic encephalopathy
11.	Severe kidney disease (as defined by estimated glomerular filtration rate <30 ml/minutes/1.73 m^2^)
12.	Known hepatitis B, hepatitis C or human immunodeficiency virus infection
13.	Patients with a history of TEE such as deep vein thrombosis, pulmonary embolism, myocardial infarction, ischemic stroke, transient ischemic attack, peripheral artery disease (Fontaine IV)
14.	Body mass index ≥40 kg/m^2^
15.	Medical conditions whose symptoms and effects could alter protein catabolism and/or IgG utilization (e.g., protein-losing enteropathies, nephrotic syndrome)
16.	Known IgA deficiency with antibodies to IgA
17.	History of hypersensitivity, anaphylaxis or severe systemic response to immunoglobulin, blood or plasma derived products or any component of Octagam 10%
18.	Known blood hyperviscosity, or other hypercoagulable states
19.	Patients with a history of drug abuse within the past five years prior to study enrollment
20.	Patients unable or unwilling to understand or comply with the study protocol
21.	Participating in another interventional clinical study with investigational treatment within three months prior to study enrollment
22.	Women who are breast feeding, pregnant, or planning to become pregnant, or are unwilling to apply an effective birth control method (such as implants, injectables, combined oral contraceptives, some intrauterine devices, sexual abstinence or vasectomized partner) up to four weeks after the last infusion received
23.	Patients who are accommodated in an institution or care facility based on an official directive or court order
24.	Patients who are in any way dependent on the sponsor, investigator or study site
25.	Patients who received forbidden medication within the washout period

Concomitant standard of care treatment including immunosuppressive drugs, corticosteroids and hydroxychloroquine were allowed, provided that treatment was initiated at least 3 months before enrollment and was set at a stable dose not exceeding the maximal dose specified in Table 2 for at least 4 weeks before study enrollment. Otherwise, medications were to be washed out according to specified wash-out periods (Table 2). Nonsteroidal anti-inflammatory drugs and opioids were allowed, provided that the treatment regimen was stable from the 2 weeks prior to study enrollment until the end of the First Period. Physical therapy-directed exercise regimens were allowed if started ≥4 weeks prior to study enrollment and kept on a stable schedule, frequency and extent until the end of the First Period. All patients were required to have muscle weakness of MMT-8 score <142, with at least 2 other abnormal CSMs.

**Table 2:** Wash out periods and maximally allowed stable doses of concomitant therapy^∗^.

Drug	Wash out period	Maximally allowed stable dose
Methotrexate	8 weeks	25 mg/week
Azathioprine	8 weeks	2 mg/kg
Cyclosporine	8 weeks	2 mg/kg
Tacrolimus	8 weeks	0.2 mg/kg
Mycophenolate mofetil	8 weeks	3000 mg daily
Leflunomide	3 months	20 mg daily
Hydroxychloroquine	8 weeks	400 mg daily
Corticosteroids	8 weeks	20 mg daily prednisone equivalent
Monoclonal antibodies (e.g. dalimumab, infliximab, ertolizumab, golimumab, abatacept, tocilizumab)	8 weeks	Not permitted
Rituximab	12 or 6 months plus normal CD19 count	Not permitted
Cyclophosphamide	3 months	Not permitted
Immunoglobulin G	6 months	Not permitted
Etanercept	4 weeks	Not permitted
Anakinra	2 weeks	Not permitted
Rilanocept	8 weeks	Not permitted
Topical steroids	2 weeks	Not permitted

As a precautionary measure, TEE prophylaxis, administered according to the standard of care, was permitted when deemed necessary by the investigator, to alleviate the elevated risk of TEEs inherent in DM patients.^[11]^

In general, premedication to alleviate potential side effects was prohibited, and could only be given if a patient experienced 2 consecutive infusion-related adverse events (AEs) that were likely to be prevented by antipyretics, antihistamines, mild analgesics, or antiemetic drugs.

Rescue treatment was not defined in the protocol. Patients receiving an excluded concomitant treatment for DM (including corticosteroid regimens beyond the permitted doses) were considered protocol deviations. In case of a major protocol deviation, the Medical Monitor decided on the further participation of the patient in the study after discussing all relevant aspects with the Investigator.

### 2.3 Arms and interventions

During the First Period (from week 0 to 16), patients received 4 cycles of either 2.0 g/kg body weight (20 ml/kg) IVIg (Octagam 10%) or placebo (20 ml/kg 0.9% w/v isotonic sodium chloride solution), administered every 4 weeks using the same infusion volumes and infusion rates. Each infusion was administered over 2 to 5 days, and the content of each infusion bag was concealed according to the blinding procedure specified in the protocol.

During the First Period, if a patient met confirmed deterioration criteria (see *Endpoints and Definitions*) at or after week 8, patients switched treatment groups while maintaining the blinding, i.e., patients in the placebo group were switched to IVIg and vice versa. Patients who received IVIg at any point in the First Period (regardless of whether they were started on IVIg or were started on placebo but then switched to IVIg) and who experienced confirmed deterioration at any time point from week 8 up to and including week 16, were to be discontinued from the study at the end of First Period. All other patients (those who received placebo throughout the First Period or those who received IVIg and did not deteriorate) were to continue into the Extension Period (weeks 16 to 40).

All patients received study medication (2.0 g/kg IVIg) in the Extension Period. If the patient was stable or improving on 2.0 g/kg, the investigator could reduce the dose of IVIg to 1.0 g/kg starting at week 28. Any patient with confirmed deterioration in the Extension Period was to discontinue the study.

### 2.4 Blinding

To maintain blinding in the First Period, infusions were given in blinded infusion bags (corresponding to a volume of 0.4 to 1.0 g/kg IVIg or sodium chloride 0.9% w/v solution). The original label was discarded together with the vial/bottle, and new label was fixed onto an opaque over-pouch by the hospital pharmacist/designee. The over-pouch was put over the infusion bag to maintain blinding. The new labels were identical for both IVIg and sodium chloride 0.9% w/v solution, so that the content of the bags was known only to the unblinded hospital pharmacist/designee. To further assure the double-blind character of this study, the investigator who administered the medication to the patient was not involved in any patient evaluation (e.g., CSM, CDASI).

The patients were blinded with respect to the treatment they received during the First Period throughout the study.

### 2.5 Endpoints and definitions

The primary endpoint of this study was the proportion of responders in the 2.0 g/kg IVIg and placebo arms at week 16. A responder was defined as a patient with a total improvement score (TIS) ≥20 (at least minimal improvement^[8]^) at week 16 and no confirmed deteriorations up to and including week 16. TIS was calculated as per 2016 American College of Rheumatology/European League Against Rheumatism (ACR/EULAR) Myositis Response Criteria for Adult DM and polymyositis.^[8]^ Patients discontinuing from the study prior to and including week 16 or with confirmed deterioration, and patients with no response (TIS <20) at week 16, were classified as non-responders.

Secondary endpoints included the proportion of responders by improvement category [minimal (TIS ≥20), moderate (TIS ≥40), major (TIS ≥60)] as per ACR/EULAR Myositis Response Criteria^[8]^ at week 16 and week 40, mean change from Baseline to end of First Period and from end of First Period to end of Extension Period in modified CDASI,^[12]^ mean change from Baseline to end of First Period and to end of Extension Period in SF-36 and 6 individual CSMs used for TIS calculation, mean change in TIS from Baseline to end of First Period and to end of Extension Period, time to minimal, moderate and major improvement as per ACR/EULAR Myositis Response Criteria,^[8]^ time to confirmed deterioration in the First Period and overall, and the proportion of patients in each treatment arm who met confirmed deterioration criteria up to and including the First Period.

Deterioration of symptoms was defined according to the Rituximab in Myositis [RIM] clinical study (excluding enzymes, as enzyme levels were not immediately available at the time of assessment), as worsening of ≥2 cm in the Physician's Global Disease Activity (GDA) on a 10 cm visual analog scale (VAS) and worsening of ≥20% in manual muscle testing (MMT)-8, or a global extra-muscular activity worsening of ≥2 cm on the myositis disease activity assessment tool (MDAAT) 10 cm VAS, or worsening by ≥30% in any 3 of 5 CSMs.^[7]^ Deterioration was considered confirmed if worsening was observed on 2 consecutive visits.

### 2.6 Primary outcome measures

The primary outcome measure is based on the TIS, as defined in *Endpoints and definitions.*^[8]^ TIS is scored on a scale of 1 to 100, and encompasses composite response criteria consisting of 6 individual CSMs: Physician's GDA; MMT-8; extra-muscular disease activity; muscle enzymes, such as aldolase, creatine kinase, alanine aminotransferase (ALT), aspartate aminotransferase (AST), and lactate dehydrogenase (LDH); Patients GDA; and Health Assessment Questionnaire – disability index (HAQ-DI) (Table 3).^[8]^ The individual CSMs of myositis disease activity have been established and validated by the International Myositis Assessment and Clinical Studies (IMACS) group and confirmed for DM in clinical studies.^[13–15]^ Recently, these 6 CSMs have been further developed by an extensive data- and international consensus-driven exercise, resulting in conjoint analysis based hybrid response criteria that combine the 6 CSMs to determine clinically meaningful improvement in the TIS.^[8]^Table 4 shows an overview of how the different CSMs are rated and the TIS calculated.

**Table 3:** Core set measures definitions used in the study.

Core set measures	
1.	Physician's GDA assessed on a 10 cm VAS as part of the MDAAT (“No evidence of disease activity” to “Extremely active or severe disease activity”)
2.	Patient's GDA assessed by the patient on a 10 cm VAS (“No evidence of disease activity” to “Extremely active or severe disease activity”)
3.	MMT-8, a set of 8 designated muscles tested bilaterally
4.	Extra-muscular activity as part of MDAAT assessed by the investigator on a VAS (a combined tool that captures the physician's assessment of disease activity of various organ systems using a scale from 0 = “Not present in the last 4 weeks” to 4 = “New - in the last 4 weeks compared to the previous 4 weeks)
5.	HAQ assessed by the patient comprised of 8 sections concerning dressing, arising, eating, walking, hygiene, reach, grip, and activities. Scoring within each section is rated from 0 (without any difficulty) to 3 (unable to do so)
6.	Enzymes worsening, aldolase, creatine kinase, ALT, AST, LDH

**Table 4:** An overview of ratings of CSM and TIS score calculation.

Core set measure	Level of improvement	Level score
Physician's global disease activity	Worsening to 5% improvement	0
	>5% to 15% improvement	7.5
	>15% to 25% improvement	15
	>25% to 40% improvement	17.5
	>40% improvement	20
Patient's global disease activity	Worsening to 5% improvement	0
	>5% to 15% improvement	2.5
	>15% to 25% improvement	5
	>25% to 40% improvement	7.5
	>40% improvement	10
Manual muscle testing-8	Worsening to 2% improvement	0
	>2% to 10% improvement	10
	>10% to 20% improvement	20
	>20% to 30% improvement	27.5
	>30% improvement	32.5
Health assessment questionnaire	Worsening to 5% improvement	0
	>5% to 15% improvement	5
	>15% to 25% improvement	7.5
	>25% to 40% improvement	7.5
	>40% improvement	10
Enzymes	Worsening to 5% improvement	0
	>5% to 15% improvement	2.5
	>15% to 25% improvement	5
	>25% to 40% improvement	7.5
	>40% improvement	7.5
Extra-muscular activity	Worsening to 5% improvement	0
	>5% to 15% improvement	7.5
	>15% to 25% improvement	12.5
	>25% to 40% improvement	15
	>40% improvement	20

### 2.7 Secondary outcome measures

Effects of IVIg on skin outcomes were evaluated using the modified CDASI, a clinician-scored, single-page instrument that separately measures activity and damage in the skin of DM patients. The modified CDASI evaluates erythema, scale, erosion/ulceration, degree of poikiloderma, and degree of calcinosis. In addition, the activity of erythema in ulceration, dyspigmentation or scarring of Gottrons papules, and changes in periungual alopecia were assessed.^[16]^ Changes in quality of life were measured using SF-36, a multi-purpose short-form health survey containing an 8-scale profile of functional health and well-being scores as well as psychometrically-based physical and mental health summary measures.

### 2.8 Safety

To evaluate the safety of IVIg in patients with DM, all AEs and serious adverse events (SAEs), pregnancies, drug overdoses, interaction, medication error and any post-study SAEs were documented throughout the study and up to 4 weeks after the last administration of IVIg or placebo. AE/SAEs were reported according to GCP guidelines of the International Council for Harmonization (ICH), with clinical site investigators determining their relatedness to the study drug as probably, possibly, unlikely related, or unrelated. (TEEs and hemolytic transfusion reactions were analyzed as AEs of special interest. Vital signs, such as blood pressure, heart rate, body temperature, and respiratory rate, as well as laboratory parameters were assessed at every visit. A physical examination was done at the Screening Visit, weeks 4, 16 and 28, and the final assessment visit (week 40). Any fatality due to AEs occurring within 4 weeks after the last drug administration was to be fully documented regardless of whether it was considered related to treatment.

### 2.9 Statistical methods

The statistical analysis was delegated to an independent external contract research organization. The sample size calculation was based on the target parameters for the evaluation of the primary endpoint. A total sample size of 84 patients was required to show a significant difference in the proportion of responders between IVIg and placebo group with a power of 80%, under the assumption that the true proportions of responders were 0.6 in the IVIg group and 0.3 in the placebo group. To allow for additional safety margin with respect to unexpected discontinuations and the use of a stratified analysis, it was planned to enroll 94 evaluable patients. The sample size calculation was based on Pearsons Chi Squared test using a two-sided alpha level of 0.05.

The primary analysis was the comparison between IVIg and placebo on the basis of the efficacy measures assessed at week 16. All analyzes were planned for the intention-to-treat as well as per-protocol sets. To evaluate the sustained benefit of treatment with IVIg and the safety and tolerability of IVIg in patients with DM, all data collected during First Period and Extension Period will be assessed.

In addition to the confirmatory evaluation of the primary endpoint, descriptive baseline summaries will be presented for each of the primary and secondary target variables. The proportion of responders within both treatment groups at week 16 will be compared using the Cochran-Mantel-Haenszel test, with a two-sided alpha level of 0.05. An exact two-sided 95% confidence interval (CI) will be constructed for the overall difference in the proportion of responders between IVIg and placebo group. In a sensitivity analysis for the primary endpoint, a logistic regression model will be applied to determine covariates, such as baseline TIS, baseline GDA and treatment. Other efficacy endpoints will be presented using descriptive statistics and inferential analyses. To confirm the sustained benefit of treatment with IVIg, response rates at week 40 and changes from week 0 to week 40 will be presented descriptively, including 95% CIs.

The proportion of patients with at least moderate and the proportion of subjects with major improvement as per ACR/EULAR Myositis Response Criteria (based on TIS) at week 16 will be calculated together according to counts and a two-sided 95% CI for the difference in the proportion of patients with improvement will be calculated. Moreover, both treatment groups will be compared using a Cochran-Mantel-Haenszel test according to improvement category, analogous to the primary analysis. The proportion of patients in each treatment arm who meet confirmed deterioration criteria up to week 16 will be presented in the same way. Changes in all secondary endpoints will be presented descriptively for First Period and Extension Period. Additionally, for all continuous secondary endpoints, an analysis of covariance (ANCOVA) will be used to analyze changes from week 0 to week 16. For patients who are switched to the alternate treatment before week 16, the last value prior to switch will be carried forward to week 16 and used to calculate change from week 0 to week 16. The model will include treatment and GDA as a fixed factor, center as random factor and the baseline value as a covariate. Least square means and 95% CI will be derived by treatment group as well as for the overall difference in the least square means between IVIg and placebo group. To assess sensitivity of the analyses, a modified ANCOVA model will be used, where the changes from week 0 to week 16 are calculated based on week 16 values, even if a patient is switched to the alternate treatment before week 16. To incorporate the switch into the model, the variable crossover (yes/no) which indicates if there was a switch to the alternate treatment will be included as additional factor in the ANCOVA model, as well as the treatment-by-crossover interaction term. In case the proportion of patients with missing values is greater than 10%, or if the modified ANCOVA detailed above indicates substantial divergence, the data will be further reviewed and analyzed.

The time to at least minimal, moderate and major improvement in TIS, and the time to confirmed deterioration will be summarized using Kaplan-Meier estimates. For analysis of outcome variables during the First Period, the time to event will be censored at the time of switch to the alternate treatment group. A stratified log-rank test will be applied for time to event variables to compare both treatment groups. Cox regression models may be applied additionally to account for randomization and other baseline covariates in the analysis.

The safety analysis will comprise descriptive statistics.

In general, missing data will not be imputed, with a few exceptions. For ANCOVA analysis of changes from baseline to week 16, the last observed value will be used in main model in case of missing values (e.g., due to early termination) and in case of switch to the alternate treatment group (as values obtained after the switch will not be included in the analysis). For missing body weight measurements, the last available measurement will be used for all calculations related to dosing.

## 3 Discussions

DM is a rare and heterogeneous disease, and management of patients with DM is challenging and evolving. The standard first-line therapy in patients with DM includes corticosteroids with or without immunosuppressants.^[1]^ Currently, IVIg is not approved by the US Food and Drug Administration (FDA) or European Medicines Agency (EMA) for treatment of DM, although its off-label use as adjuvant therapy is widespread.^[17]^ However, due to lack of regulatory approval, many patients are not able to get insurance coverage for IVIg, or they require failure of several other immunosuppressive drugs. Therefore, IVIg is mainly used when treatment with glucocorticoids and/or other immunosuppressive agents does not produce a sufficient improvement in disease.

IVIg is commonly used as a corticosteroid-sparing agent and represents a unique treatment option for DM patients with active infection or at high risk of infection. IVIg therapy may present a safe and effective treatment option, especially in patients showing no response or incomplete response to corticosteroids or who experience severe side effects on these medications.^[17,18]^

There is a general lack of data in DM from large, randomized, controlled clinical studies with validated measurable outcomes and long-term follow-up. Building on the experience with off-label use of IVIg in DM, the ProDERM study aims to provide important information on efficacy and safety of IVIg (Octagam 10%) in a large, placebo-controlled, randomized, clinical study.

The ProDERM study enrolled a relatively large number of patients for a rare disease and had a unique design to allow a treatment switch for deteriorating patients in the randomized study phase. This allowed for better recruitment and safety of the patients, without compromising study blinding in the study. The length of treatment in the placebo-controlled phase was 4 courses of IVIg, with the primary endpoint evaluated after 16 weeks of treatment. In the 1993 study by Dalakas et al, patients responded after only 2 courses of IVIg.^[6]^ However, based on clinical experience, the Steering Committee recommended to extend the primary efficacy observation period to 16 weeks. All eligible patients were treated with IVIg for a further 24 weeks (6 months), as recommended by the IMACS, to gather evidence on long-term use of IVIg in DM patients.^[19]^

The outcome measures in the ProDERM study include robust response criteria of myositis disease activity that have been established by the IMACS, approved by ACR/EULAR, and validated in clinical studies with DM patients.^[13–15]^ The primary outcome measure used was developed by extensive data and consensus methodology using conjoint analysis, enabling the assessment of clinically meaningful improvements in various categories (minimal, moderate, and major improvement) as well as a continuous score for the total magnitude of improvement (as TIS).^[8,13–15]^ Another large, randomized, controlled study in patients with DM, the RIM study, employed similar CSMs in their definition of improvement (DOI).^[7]^ However, the definition used in the RIM study required a minimal severity baseline deficit of at least 20% in each CSM in the clinical trial inclusion criteria to enable reaching the threshold of ≥20% improvement in CSM after treatment. This rendered it difficult for enrollment of mild or moderate phenotypes. Moreover, the DOI had other limitations of using the same weight for all CSMs and had only one category of response: improved or not, disregarding the magnitude of improvement. The ACR/EULAR Myositis Response Criteria with TIS^[8]^ used in the ProDERM study included CSMs that are weighted, with both subjective and measurable outcomes, and can be used as continuous or categorical response criteria. The use of a TIS allows the ProDERM study to assess the full range of response from minimal to major, which is important for its applicability in the clinic.^[8]^ Another advantage of this TIS over the previous DOI is that it enabled inclusion of patients with milder phenotype (mild to moderate disease severity) to evaluate the response.^[16]^

It is important to consider that certain standard of care medications for DM (i.e., steroids, hydroxychloroquine, and immunosuppressants) were permitted during the study, provided that the treatment was initiated 3 months prior to randomization and set at a stable dose. This means that a high level of deterioration is not expected in the placebo group. While this might make it more difficult to observe improvement with IVIg compared with placebo, it is reflective of the current recommendations for the use of IVIg as well as current clinical practice.^[2–4]^

Patients in this study were treated with high-dose (2.0 g/kg) IVIg for up to 40 weeks. The investigators had the opportunity to decrease the dose to 1.0 g/kg from week 28 onwards if the patients condition allowed, following a recommendation of the FDA. Due to the use of long-term IVIg treatment at a potentially high dose in this study, and as patients with DM are at higher risk of developing TEEs than the general population,^[11,20]^ particular emphasis was placed on monitoring for TEEs and hemolytic transfusion reactions.

This is the first prospective multicenter study to evaluate the long-term efficacy and safety of IVIg in a placebo-controlled, randomized setting for patients with DM. The ProDERM study aims to provide insights that could inform on treatment decisions, with the hope of offering DM patients an additional effective and well-tolerated maintenance therapy option.

## Author contributions

**Conceptualization:** Rohit Aggarwal, Christina Charles-Schoeman, Joachim Schessl, Mazen M. Dimachkie, Irene Beckmann, Todd Levine.

**Methodology:** Rohit Aggarwal, Christina Charles-Schoeman, Joachim Schessl, Mazen M. Dimachkie, Irene Beckmann, Todd Levine.

**Writing – original draft:** Rohit Aggarwal.

**Writing – review & editing:** Rohit Aggarwal, Christina Charles-Schoeman, Joachim Schessl, Mazen M. Dimachkie, Irene Beckmann, Todd Levine.
